# Interlaminar Versus Transforaminal Full-Endoscopic Lumbar Discectomy for L4–L5 Disc Herniation: Operative Time, Radiation Exposure, Complications, and Learning Curve

**DOI:** 10.7759/cureus.100376

**Published:** 2025-12-29

**Authors:** Tomasz Sienkiel, Marcin Gaska, Jakub Kalisz, Przemyslaw Koszyk, Barbara Jasiewicz

**Affiliations:** 1 Department of Orthopedics, University Orthopedic and Rehabilitation Hospital, Zakopane, POL; 2 Department of Orthopedics, Jagiellonian University Medical College, Krakow, POL

**Keywords:** conscious analgosedation, fluoroscopy exposure, full-endoscopic lumbar discectomy, interlaminar approach, l4–l5 disc herniation, learning curve, operative outcomes, transforaminal approach

## Abstract

Background

L4-L5 disc herniation is a common cause of radiculopathy, yet the optimal full-endoscopic lumbar discectomy approaches: interlaminar endoscopic lumbar discectomy (IELD) or transforaminal endoscopic lumbar discectomy (TELD), remain debated due to level-specific anatomical constraints. This study compared operative efficiency, radiation exposure, complications, and clinical outcomes between IELD and TELD at the L4-L5 level.

Methods

A retrospective analysis of 131 consecutive L4-L5 full-endoscopic discectomies (42 IELD, 89 TELD) performed between 2021 and 2023 was conducted. Operative time, fluoroscopic dose-area product, anesthesia type, complications, and patient-reported outcomes (VAS, ODI) were recorded at baseline, 3 months, and 12 months. Temporal trends and learning-curve patterns were evaluated.

Results

A marked shift from IELD to TELD occurred over the study period, with TELD accounting for 90% of cases by 2023, and conscious analgesia was used in 94%. Operative time improved for both techniques, plateauing at 47 minutes for IELD and 42 minutes for TELD. Radiation exposure declined substantially for both approaches, decreasing from 160 to 120 µGym² for IELD and from 280 to 180 µGy·m² for TELD; however, TELD remained numerically higher than IELD in the late phase. All complications (five total) occurred early in the study period, with none recorded after 2022. Both approaches produced significant improvements in VAS and ODI scores, with comparable long-term clinical outcomes between groups.

Conclusions

Both IELD and TELD produced significant and sustained improvements in VAS and ODI, with no differences in long-term clinical outcomes. TELD showed a steeper learning-curve pattern and shorter operative times after proficiency was achieved, and it was increasingly performed under conscious analgesia. Fluoroscopy dose declined substantially over time for both approaches; however, TELD required higher radiation exposure during early adoption and remained numerically higher than IELD in late phases. Approach selection at L4-L5 should therefore prioritize anatomy, surgeon experience, and perioperative goals rather than expectations of superior long-term outcomes.

## Introduction

Lumbar disc herniation at the L4-L5 level is one of the most common causes of radiculopathy and functional disability in adults and accounts for a substantial proportion of surgical interventions for lumbar disc disease [1,2]. This motion segment is biomechanically distinct, characterized by high mobility and increased shear forces, which predispose it to degenerative changes and symptomatic disc herniation [3]. These anatomical and mechanical features directly influence surgical access, technical complexity, and the risk profile of different operative approaches.

Over the past two decades, full-endoscopic lumbar discectomy has become a well-established minimally invasive alternative to open and microdiscectomy techniques, offering comparable clinical outcomes with reduced tissue trauma and faster recovery [4]. Two principal endoscopic approaches are currently used: interlaminar endoscopic lumbar discectomy (IELD) and transforaminal endoscopic lumbar discectomy (TELD).

The interlaminar approach follows a posterior midline trajectory and provides excellent exposure at the L5-S1 level, where the interlaminar window is wide [5]. Conversely, the L4-L5 segment presents a constricted interlaminar window, which limits the ergonomic corridor and necessitates greater neural manipulation, potentially increasing the risk of nerve irritation and prolonging operative time, particularly during the early learning phase [5].

The transforaminal approach accesses the disc through Kambin’s triangle, allowing decompression while minimizing dural sac and nerve root retraction and preserving posterior stabilizing structures [6,7]. At the L4-L5 level, foraminal anatomy often provides a favorable working corridor, making TELD particularly suitable for this segment [7]. An additional advantage of TELD is the feasibility of performing the procedure under conscious anglosedation, enabling real-time neurologic feedback and early detection of nerve irritation, thereby enhancing intraoperative safety [8].

A recognized limitation of TELD is its greater reliance on fluoroscopic guidance during the early learning phase, which may result in temporarily higher radiation exposure [9]. However, previous studies have shown that fluoroscopic use decreases substantially with increasing surgeon experience, reflecting a steep learning curve associated with transforaminal endoscopic techniques [10,11].

Despite the widespread use of both approaches, high-quality level-specific comparative data for L4-L5 disc herniation remain limited. Most published studies analyze mixed lumbar levels or focus on other segments, restricting their applicability to level-specific surgical decision-making at L4-L5 [12-15]. Consequently, the optimal full-endoscopic approach for this anatomically unique level remains debated.

Between 2021 and 2023, our institution transitioned from predominantly using IELD to the routine application of TELD for L4-L5 disc herniation, accompanied by increasing surgical experience and progressive adoption of conscious anglosedation. This evolution provided a unique opportunity to evaluate level-specific differences between approaches in operative efficiency, radiation exposure, complication patterns, and learning-curve dynamics.

The aim of this study was to compare interlaminar and transforaminal full-endoscopic approaches for the treatment of L4-L5 disc herniation. We hypothesized that, after completion of the learning curve, TELD would demonstrate improved operative efficiency and perioperative safety while maintaining long-term clinical outcomes equivalent to those achieved with IELD.

## Materials and methods

Study design and population

A retrospective analysis was performed on 131 consecutive patients who underwent full-endoscopic lumbar discectomy at the L4-L5 level between January 2021 and November 2023 at a single university orthopedic center. All procedures were performed by the same spine surgeon. Clinical and operative data were prospectively collected and subsequently analyzed retrospectively after completion of follow-up.

Inclusion and exclusion criteria

Patients were eligible for inclusion if they had a single-level L4-L5 lumbar disc herniation confirmed on magnetic resonance imaging, radiological findings consistent with radicular pain or neurological deficit, failure of adequate conservative treatment, and complete clinical and radiographic documentation.

Patients were excluded if they had recurrent or multi-level lumbar disc herniation, radiographic evidence of lumbar instability on flexion-extension radiographs, a history of prior lumbar fusion surgery, incomplete clinical data, or lack of consent for use of medical records.

Study groups

Patients were divided into two groups according to the surgical approach used. The first group underwent interlaminar endoscopic lumbar discectomy (IELD), while the second group underwent transforaminal endoscopic lumbar discectomy (TELD). Baseline demographic and clinical characteristics of both groups were comparable. The type of anesthesia used for each procedure, either general anesthesia or conscious analgosedation, was documented.

Surgical techniques

Interlaminar endoscopic lumbar discectomy was performed with the patient in the prone position under fluoroscopic localization of the interlaminar window. After identification of the target level, the working cannula was advanced through a split in the ligamentum flavum to access the spinal canal. Herniated disc fragments were removed under direct endoscopic visualization.

Transforaminal endoscopic lumbar discectomy (TELD) was performed using a lateral entry point approximately 10-12 cm from the midline under fluoroscopic guidance. After skin incision, sequential dilation was used to access the neuroforamen through Kambin’s triangle.

A form of foraminoplasty was routinely performed in all TELD cases as part of the standard access technique, using sequential reamers that resulted in minimal to moderate enlargement of the neural foramen to allow safe placement of the working cannula. The extent of foraminoplasty was graded rather than binary, with additional bone removal performed when required based on preoperative imaging and intraoperative anatomical conditions, including foraminal stenosis, facet joint hypertrophy, a high iliac crest, or a restricted working corridor.

When necessary, extended foraminoplasty was performed using endoscopic reamers or burrs to further optimize foraminal dimensions and ensure atraumatic access to the herniated disc. Herniated disc fragments were removed under direct endoscopic visualization with continuous saline irrigation. TELD procedures were initially performed under general anesthesia and were later transitioned to conscious analgosedation to allow real-time neurological feedback during decompression. All procedures were performed using a 7-mm endoscope (Richard Wolf, Germany) with continuous saline irrigation.

Data collection and outcome measures

Collected variables included surgical approach, anesthesia modality, operative time measured from skin incision to closure, and fluoroscopic radiation exposure recorded as dose-area product (µGym²). Intraoperative and postoperative complications, including nerve irritation, dural tear, hematoma, and infection, were documented.

Pain intensity was assessed using the Visual Analog Scale (VAS) [16], and functional disability was evaluated using the Oswestry Disability Index (ODI), using the validated Polish version [17], both of which are validated instruments freely available for clinical and academic research use. Clinical outcomes were recorded preoperatively and at 3-month and 12-month follow-up visits.

Radiation exposure assessment

Radiation exposure was obtained directly from the fluoroscopy system console as the dose-area product. Surgeon radiation exposure was estimated using previously published conversion factors for endoscopic spine procedures.

Statistical analysis

Continuous variables were expressed as mean ± standard deviation or median with range, depending on data distribution. Categorical variables were reported as absolute numbers and percentages. Between-group comparisons were performed using the Student’s t-test or Mann-Whitney U test for continuous variables and the chi-square test for categorical variables. Temporal trends and learning-curve effects were assessed using linear regression analysis. A p-value of less than 0.05 was considered statistically significant. Statistical analyses were performed using SPSS software (IBM Corp., Armonk, NY).

Ethics approval

This study was approved by the Institutional Review Board of the Jagiellonian University Medical College (protocol number 1072.6120321.2021) and was conducted in accordance with the Declaration of Helsinki. Due to the retrospective design of the study, the requirement for informed consent was waived.

## Results

A total of 131 patients underwent full-endoscopic lumbar discectomy at the L4-L5 level between January 2021 and November 2023. Forty-two patients were treated using the interlaminar approach (IELD) and 89 using the transforaminal approach (TELD). Baseline demographic and preoperative clinical characteristics were comparable between groups and are summarized in Table 1.

**Table 1:** Baseline Demographic and Clinical Characteristics of Patients Undergoing IELD and TELD at L4–L5. Demographic and preoperative clinical variables were comparable between groups, with no significant differences in age, sex, BMI, symptom duration, or baseline pain and disability scores. A significant difference was observed in anesthesia modality, reflecting the transition to conscious analgosedation during the TELD learning curve. Continuous variables were compared using the Student’s t-test. Categorical variables were compared using the chi-square (χ²) test. IELD: Interlaminar Endoscopic Lumbar Discectomy; TELD: Transforaminal endoscopic lumbar discectomy; VAS: Visual Analog Scale [16]; ODI: Oswestry Disability Index [17].

Variables	IELD (n = 42)	TELD (n = 89)	Statistical test	Test statistic	p-value
Age (years), mean ± SD	48.6 ± 12.3	49.8 ± 11.7	Student’s t-test	t = 0.61	0.54
Sex (male), n (%)	22 (52%)	45 (51%)	χ² test	χ² = 0.01	0.91
BMI (kg/m²), mean ± SD	27.3 ± 3.4	27.8 ± 3.1	Student’s t-test	t = 0.70	0.48
Symptom duration (months), mean ± SD	5.1 ± 2.4	4.8 ± 2.1	Student’s t-test	t = 0.90	0.37
Motor deficit, n (%)	7 (17%)	13 (15%)	χ² test	χ² = 0.08	0.78
Positive straight-leg raise, n (%)	36 (86%)	78 (88%)	χ² test	χ² = 0.11	0.74
Preoperative VAS-leg (0–10), mean ± SD	7.3 ± 1.1	7.5 ± 1.2	Student’s t-test	t = 0.86	0.39
Preoperative ODI (%), mean ± SD	33.1 ± 6.8	33.6 ± 7.1	Student’s t-test	t = 0.43	0.67
General anesthesia, n (%)	42 (100%)	54 (61%)	χ² test	χ² = 27.9	<0.001
Conscious analgosedation, n (%)	0 (0%)	35 (39%)	χ² test	χ² = 27.9	<0.001

Distribution of surgical techniques and anesthesia

A clear procedural transition was observed over the study period. TELD accounted for 33% of L4-L5 procedures in 2021 and progressively increased to 90% by 2023. In parallel, the use of conscious analgosedation increased from 0% during the early study phase to 94% in 2023, reflecting growing surgeon experience and reduced reliance on general anesthesia.

Operative and perioperative parameters for both approaches are summarized in Table 2.

**Table 2:** Operative and Perioperative Parameters for IELD and TELD at the L4–L5 Level. TELD demonstrated significantly shorter operative times after completion of the learning curve and initially required higher fluoroscopic exposure, which decreased substantially with surgeon experience. IELD showed slightly lower radiation exposure during early phases but a higher rate of early neurological irritation. Hospital length of stay was identical in both groups due to mandatory reimbursement criteria of the national health system (NFZ).

Parameters	IELD (n = 42)	TELD (n = 89)	Statistical test	Test statistic	p-value
Operative time (min), late phase/plateau, mean ± SD	47 ± 9	42 ± 8	Student’s t-test	t = 2.61	0.01
Operative time (min), early phase, mean ± SD	65 ± 12	70 ± 14	Student’s t-test	t = 1.56	0.12
Fluoroscopy dose (DAP, µGy·m²), late phase/plateau, median	160	180	Mann–Whitney U test	U = 1358	0.04
Fluoroscopy dose (DAP, µGy·m²), early phase, median	160	280	Mann–Whitney U test	U = 624	<0.001
Overall complication rate, n (%)	4 (9.5%)	1 (1.1%)	Chi-square (χ²) test	χ² = 4.70	0.03
Recurrent disc herniation, n (%)	2 (4.7%)	5 (5.6%)	Chi-square (χ²) test	χ² = 0.05	0.83
Anesthesia modality, n (%)	42 (100%) general anesthesia	54 (61%) general anesthesia; 35 (39%) analgosedation	Chi-square (χ²) test	χ² = 27.9	<0.001

Operative time

Both IELD and TELD demonstrated learning-curve-related reductions in operative time. In the IELD group, mean operative time decreased from approximately 65 minutes during the early phase to 55 minutes in the mid-phase and reached a plateau of 47 minutes after approximately 30 cases. In the TELD group, operative time initially averaged 70 minutes during early adoption, improved to approximately 55 minutes during the mid-phase, and reached a plateau of 42 minutes after approximately 60 cases. By the final study year, TELD procedures were consistently shorter than IELD procedures (Table 2).

Radiation exposure

Fluoroscopic radiation exposure demonstrated a progressive decline for both techniques over time. Year-by-year fluoroscopic dose-area product (DAP) values are presented in Table 3. In the IELD group, median DAP decreased from 160 µGym² in 2021 to 150 µGym² in 2022 and further to 120 µGym² in 2023. In contrast, TELD initially required substantially higher fluoroscopic exposure, with a median DAP of 280 µGym² in 2021 during the early learning phase. However, radiation exposure declined sharply with experience, decreasing to 220 µGym² in 2022 and reaching 180 µGym² in 2023. Although TELD remained numerically associated with higher radiation exposure than IELD in the late phase, both approaches ultimately achieved clinically acceptable low-dose radiation levels.

**Table 3:** Fluoroscopic Radiation Dose (DAP, µGy·m²) by Year for IELD and TELD Table presents year-by-year changes in fluoroscopic radiation dose expressed as dose–area product (DAP). TELD was associated with significantly higher radiation exposure during the early learning phase (2021), followed by a marked and progressive reduction with increasing surgeon experience. By 2023, both IELD and TELD achieved low and clinically acceptable radiation levels, with TELD demonstrating the steepest reduction over time.

Year	IELD Median DAP (µGym²)	TELD Median DAP (µGym²)	Statistical test (IELD vs TELD)	Test statistic	p-value
2021	160	280	Mann–Whitney U test	U = 412	<0.001
2022	150	220	Mann–Whitney U test	U = 589	0.01
2023	120	180	Mann–Whitney U test	U = 721	0.04

Complications

All perioperative complications occurred during the early phases of the study. In the IELD group, four complications were observed, including two cases of transient nerve irritation and two cases of traversing nerve root irritation or injury. In the TELD group, one dural tear occurred during the early learning phase and was managed conservatively. No neurologic, dural, infectious, or hematoma-related complications were recorded after 2022 in either group. Recurrent disc herniation occurred in 4.7% of IELD cases and 5.6% of TELD cases, with no statistically significant difference between approaches (Table 2). Due to the low absolute number of adverse events, complication data were analyzed descriptively, and no formal statistical comparisons between approaches were performed.

Clinical outcomes

Both surgical approaches resulted in substantial and sustained improvement in pain and functional outcomes. Mean VAS-leg scores improved from approximately 7.4 preoperatively to 1.3 at 12 months, while ODI scores improved from approximately 33% preoperatively to 6-7% at 12 months (p< 0.001 for both). There were no statistically significant differences between IELD and TELD in VAS-leg or ODI scores at baseline, 3 months, or 12 months. Detailed clinical outcome data are presented in Table 4.

**Table 4:** Clinical outcomes after IELD and TELD at the L4–L5 level. Table summarizes patient-reported clinical outcomes following interlaminar (IELD) and transforaminal (TELD) full-endoscopic lumbar discectomy. Both techniques resulted in significant improvement in leg pain (VAS-leg)[16] and disability (ODI)[17] at 3 and 12 months compared with baseline. No statistically significant differences were observed between the two approaches at any evaluated time point. VAS: Visual Analog Scale; ODI: Oswestry Disability Index.

Outcome	Time point	IELD (n = 42), mean ± SD	TELD (n = 89), mean ± SD	Statistical test	Test statistic	p-value
VAS-leg (0–10)	Baseline	7.3 ± 1.1	7.5 ± 1.2	Student’s t-test	t = 0.86	0.39
	3 months	2.0 ± 0.9	1.9 ± 0.8	Student’s t-test	t = 0.55	0.58
	12 months	1.4 ± 0.7	1.3 ± 0.6	Student’s t-test	t = 0.49	0.62
ODI (%)	Baseline	33.1 ± 6.8	33.6 ± 7.1	Student’s t-test	t = 0.43	0.67
	3 months	12.4 ± 4.2	11.8 ± 4.0	Student’s t-test	t = 0.77	0.44
	12 months	7.0 ± 3.1	6.7 ± 3.0	Student’s t-test	t = 0.63	0.53

Pain intensity was assessed using the Visual Analog Scale (VAS), and functional disability was evaluated using the Oswestry Disability Index (ODI). Both instruments are validated and widely used clinical outcome measures. The VAS is freely available for clinical and research use, while the ODI was used in its original, unmodified form in accordance with its published guidelines.

Learning curve analysis

Learning-curve analysis demonstrated nonlinear improvements in operative efficiency and radiation exposure for both techniques. In the IELD group, an inflection point was observed near case 22, corresponding to a reduction in operative time from approximately 55 to 47 minutes and a reduction in median radiation dose from 180 to 120 µGym². In the TELD group, a major inflection occurred around case 25, followed by marked reductions in operative time from approximately 70 to 42 minutes and radiation exposure from 280 to 180 µGy·m². A detailed breakdown of learning-curve phases for both approaches is provided in Table 5.

**Table 5:** Learning Curve Summary for IELD and TELD at L4–L5. Learning curve phases for interlaminar (IELD) and transforaminal (TELD) full-endoscopic lumbar discectomy at the L4–L5 level. Data are presented descriptively to illustrate trends in operative time and fluoroscopic radiation dose across consecutive procedural phases. No formal statistical comparisons were performed between learning-curve phases.

Technique	Phase (case range)	Approx. cases in phase, n	Mean operative time (min)	Median radiation dose (µGy·m²)
IELD	Early (cases 1–15)	15	65	180
IELD	Mid (cases 16–30)	15	55	150
IELD	Late / plateau (cases 31–42)	12	47	120
TELD	Early (cases 1–30)	30	70	280
TELD	Mid (cases 31–60)	30	55	220
TELD	Late / plateau (cases 61–89)	29	45	180

**Figure 1:**
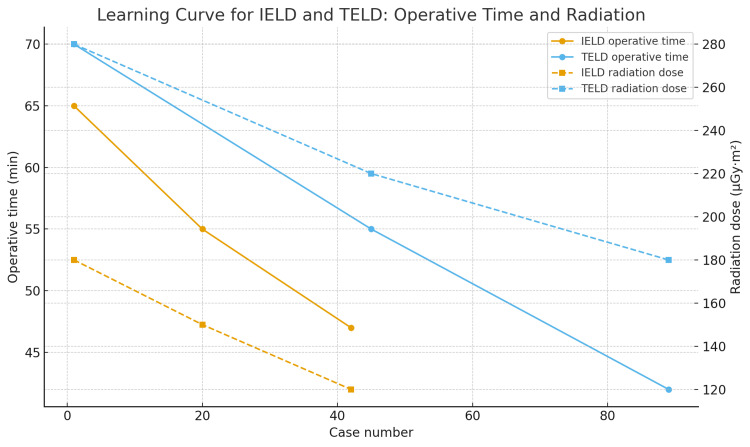
Learning curve for IELD and TELD at the L4–L5 level. Curves demonstrate progressive reduction in operative time and fluoroscopic dose with growing surgeon experience. TELD shows a steeper improvement in both parameters, particularly in radiation efficiency, despite higher initial dose.

Operative time and radiation-dose trajectories are illustrated in Figure 1.

Overall, TELD exhibited a steeper learning curve and ultimately achieved shorter operative times, lower complication rates, radiation exposure equivalent to IELD, and high feasibility under conscious analgosedation.

## Discussion

This level-specific analysis demonstrates clear differences between interlaminar (IELD) and transforaminal (TELD) full-endoscopic approaches for the treatment of L4-L5 disc herniation. Although both techniques achieved excellent long-term clinical outcomes, their intraoperative characteristics, learning-curve dynamics, and complication profiles differed substantially, reflecting the biomechanical and anatomical uniqueness of the L4-L5 motion segment.

The interlaminar approach offers a familiar posterior trajectory and provides excellent access at the L5-S1 level, where the interlaminar window is naturally wide [18]. Conversely, the L4-L5 segment presents a constricted interlaminar window, which limits the ergonomic corridor and necessitates greater nerve manipulation [19,20]. These anatomical constraints were reflected in our findings: IELD demonstrated a more gradual learning curve, with an inflection point around case 22, and early complications-including transient nerve irritation-occurred exclusively during the initial learning phase. Similar early neurologic events related to interlaminar access at upper lumbar levels have been reported previously [21].

In contrast, the transforaminal approach allows access to the disc through Kambin’s triangle while avoiding retraction of the dural sac and posterior stabilizing structures [22]. This biomechanical advantage is particularly relevant at L4-L5, where foraminal anatomy frequently provides a safe and efficient working corridor. In the present cohort, TELD exhibited a steeper and earlier learning-curve transition, with a major inflection around case 25 and rapid improvements in operative time and procedural efficiency. These observations are consistent with prior studies describing accelerated proficiency acquisition in transforaminal endoscopic surgery [23].

Although TELD initially required substantially higher fluoroscopic exposure, well-recognized limitation during the early adoption phase of transforaminal endoscopy-radiation dose decreased rapidly with increasing experience and ultimately reached clinically acceptable levels comparable to IELD. This finding confirms that the radiological disadvantage of early TELD is transient and technique-dependent rather than intrinsic to the transforaminal approach itself.

One of the most clinically meaningful advantages of TELD is its compatibility with conscious analgosedation. Awake endoscopic surgery enables real-time neurologic feedback, facilitating immediate recognition of nerve irritation and enhancing intraoperative safety [24]. In our study, all neurologic complications occurred before the routine implementation of analgosedation, and no adverse neurologic events were observed thereafter. Prior studies have also shown that awake endoscopy is associated with reduced postoperative pain, earlier mobilization, and faster functional recovery, supporting its role in patient-centered and ambulatory spine surgery pathways. From a health-care system perspective, the ability to perform TELD under conscious analgosedation may facilitate a transition toward outpatient or short-stay spine surgery models.

From an economic and logistical standpoint, full-endoscopic spine surgery requires specialized equipment and surgeon training, which may limit accessibility in some health-care settings. While both IELD and TELD utilize similar endoscopic platforms, TELD may initially incur higher costs related to fluoroscopy use and training during the learning phase. However, with increasing experience, improved operative efficiency, reduced complication rates, and the feasibility of ambulatory surgery may offset these early investments. Therefore, cost-effectiveness is likely influenced more by surgeon experience and institutional workflow than by the choice of endoscopic corridor alone.

Despite these intraoperative differences, long-term functional outcomes were equivalent between IELD and TELD in this cohort. Both approaches resulted in significant and sustained improvements in VAS and ODI scores, consistent with findings from comparative studies and meta-analyses demonstrating similar clinical effectiveness among endoscopic techniques and conventional or microdiscectomy procedures [24]. These results underscore that approach selection should be guided by anatomical considerations, surgeon experience, and perioperative goals rather than expectations of long-term clinical outcome alone. Importantly, TELD does not confer superior long-term clinical outcomes compared with IELD but becomes a more efficient and patient-centered technique after completion of the learning curve.

A key strength of this study is its strict focus on a single lumbar level. Most existing comparative analyses combine multiple lumbar segments, limiting interpretation due to the distinct morphology and biomechanics of L4-L5. The consecutive patient cohort, standardized surgical workflow, and documented transition from IELD to TELD further strengthen the internal validity of the findings. Limitations include the retrospective design and single-surgeon setting, which may limit generalizability but also minimize procedural variability. Future multicenter prospective studies incorporating surgeon-stratified learning-curve analysis are warranted to further refine level-specific approach selection.

In summary, while both IELD and TELD provide excellent long-term outcomes for L4-L5 disc herniation, their operative characteristics differ substantially. TELD begins with higher fluoroscopic demand but rapidly evolves into a highly efficient, low-radiation technique with the added benefit of safe and effective conscious analgosedation. Once proficiency is achieved, TELD emerges as the preferred full-endoscopic approach for L4-L5 disc herniation due to its shorter operative time, favorable complication profile, optimized radiation efficiency, and patient-centered perioperative advantages.

## Conclusions

Both interlaminar (IELD) and transforaminal (TELD) full-endoscopic lumbar discectomy provide excellent long-term clinical improvement for patients with L4-L5 disc herniation. However, their intraoperative performance differs substantially at this anatomically constrained level. IELD remains a viable technique, but its effectiveness at L4-L5 is limited by the narrow interlaminar window and the greater need for nerve manipulation.

TELD demonstrated a distinct and steeper learning curve, with progressive gains in operative efficiency, radiation control, and complication reduction. Although initial fluoroscopic exposure was higher and remained numerically greater than IELD in later phases, radiation use decreased markedly with experience and reached clinically acceptable low-dose levels. Importantly, TELD allows the routine use of conscious analgosedation, which enhances intraoperative safety and facilitates faster mobilization and recovery. After proficiency is achieved, TELD emerges as the preferred full-endoscopic approach for L4-L5 disc herniation, combining superior operative performance with equally favorable long-term outcomes compared with IELD.

## References

[1] Pojskic M, Bisson E, Oertel J, Takami T, Zygourakis C, Costa F (2024). Lumbar disc herniation: epidemiology, clinical and radiologic diagnosis WFNS spine committee recommendations. World Neurosurg X.

[2] Kreiner DS, Hwang SW, Easa JE (2014). An evidence-based clinical guideline for the diagnosis and treatment of lumbar disc herniation with radiculopathy. Spine J.

[3] Lakomkin N, Stannard B, Fogelson JL, Mikula AL, Lenke LG, Zuckerman SL (2021). Comparison of surgical invasiveness and morbidity of adult spinal deformity surgery to other major operations. Spine J.

[4] Ahn Y (2012). Transforaminal percutaneous endoscopic lumbar discectomy: technical tips to prevent complications. Expert Rev Med Devices.

[5] Ruetten S, Komp M, Merk H, Godolias G (2008). Full-endoscopic interlaminar and transforaminal lumbar discectomy versus conventional microsurgical technique: a prospective, randomized, controlled study. Spine (Phila Pa 1976).

[6] Ahn Y, Youn MS, Heo DH (2019). Endoscopic transforaminal lumbar interbody fusion: a comprehensive review. Expert Rev Med Devices.

[7] Khandge AV, Sharma SB, Kim JS (2021). The evolution of transforaminal endoscopic spine surgery. World Neurosurg.

[8] Yang L, Pan YL, Liu CZ, Guo DX, Zhao X (2022). A retrospective comparative study of local anesthesia only and local anesthesia with sedation for percutaneous endoscopic lumbar discectomy. Sci Rep.

[9] Choi MH, Choi BG, Jung SE, Byun JY (2016). Factors related to radiation exposure during lumbar spine intervention. J Korean Med Sci.

[10] Choi KC, Lee DC, Shim HK, Shin SH, Park CK (2017). Strategy of percutaneous endoscopic lumbar discectomy for migrated disc herniation. World Neurosurg.

[11] Ahn Y, Lee S, Son S, Kim H (2021). Learning curve for interlaminar endoscopic lumbar discectomy: a systematic review. World Neurosurg.

[12] Chen KT, Choi KC, Shim HK, Lee DC, Kim JS (2022). Full-endoscopic versus microscopic unilateral laminotomy for bilateral decompression of lumbar spinal stenosis at L4-L5: comparative study. Int Orthop.

[13] Phan K, Xu J, Schultz K (2017). Full-endoscopic versus micro-endoscopic and open discectomy: A systematic review and meta-analysis of outcomes and complications. Clin Neurol Neurosurg.

[14] Lambrechts MJ, Canseco JA, Toci GR (2023). Spine surgical subspecialty and its effect on patient outcomes: a systematic review and meta-analysis. Spine (Phila Pa 1976).

[15] Zhao Q, Xiao L, Wu Z, Liu C, Zhang Y (2022). Comparison of the efficacy of fully endoscopic spine surgery using transforaminal and interlaminar approaches in the treatment of prolapsed lumbar 4/5 disc herniation. J Orthop Surg Res.

[16] Huskisson EC (1974). Measurement of pain. The Lancet.

[17] Miekisiak G, Kollataj M, Dobrogowski J (2013). Validation and cross-cultural adaptation of the Polish version of the Oswestry Disability Index. Spine (Phila Pa 1976).

[18] Kotheeranurak V, Liawrungrueang W, Quillo-Olvera J (2023). Full-endoscopic lumbar discectomy approach selection: a systematic review and proposed algorithm. Spine (Phila Pa 1976).

[19] Hua W, Zhang Y, Wu X (2019). ull-endoscopic visualized foraminoplasty and discectomy under general anesthesia for L4-L5 and L5-S1 disc herniation. Spine (Phila Pa 1976).

[20] Kim JY, Kim HS, Wu PH (2021). Anatomical importance of inner ligamentum flavum parameters for endoscopic lumbar decompression. JMISST.

[21] Li L, Chang F, Hai Y (2020). Clinical effect evaluation and correlation between preoperative imaging parameters and clinical effect of endoscopic Transforaminal decompression for lumbar spinal stenosis. BMC Musculoskelet Disord.

[22] Tornatore I, Basile A, Aureli A, Tarantino A, Orlando G, Buharaja R (2025). Effectiveness and safety of transforaminal spinal endoscopy: analysis of 1000 clinical cases. Diagnostics (Basel).

[23] Bhaisare R, Kamble B, Patond K (2016). Long-term results of endoscopic lumbar discectomy using Destandau technique. Asian Spine J.

[24] Li WS, Yan Q, Cong L (2022). Endoscopic versus non-endoscopic discectomy for lumbar disc herniation: a systematic review and meta-analysis. Global Spine J.

